# Gene-Environment Interactions Relevant to Estrogen and Risk of Breast Cancer: Can Gene-Environment Interactions Be Detected Only among Candidate SNPs from Genome-Wide Association Studies?

**DOI:** 10.3390/cancers13102370

**Published:** 2021-05-14

**Authors:** JooYong Park, Ji-Yeob Choi, Jaesung Choi, Seokang Chung, Nan Song, Sue K. Park, Wonshik Han, Dong-Young Noh, Sei-Hyun Ahn, Jong Won Lee, Mi Kyung Kim, Sun Ha Jee, Wanqing Wen, Manjeet K. Bolla, Qin Wang, Joe Dennis, Kyriaki Michailidou, Mitul Shah, Don M. Conroy, Patricia A. Harrington, Rebecca Mayes, Kamila Czene, Per Hall, Lauren R. Teras, Alpa V. Patel, Fergus J. Couch, Janet E. Olson, Elinor J. Sawyer, Rebecca Roylance, Stig E. Bojesen, Henrik Flyger, Diether Lambrechts, Adinda Baten, Keitaro Matsuo, Hidemi Ito, Pascal Guénel, Thérèse Truong, Renske Keeman, Marjanka K. Schmidt, Anna H. Wu, Chiu-Chen Tseng, Angela Cox, Simon S. Cross, kConFab Investigators, Irene L. Andrulis, John L. Hopper, Melissa C. Southey, Pei-Ei Wu, Chen-Yang Shen, Peter A. Fasching, Arif B. Ekici, Kenneth Muir, Artitaya Lophatananon, Hermann Brenner, Volker Arndt, Michael E. Jones, Anthony J. Swerdlow, Reiner Hoppe, Yon-Dschun Ko, Mikael Hartman, Jingmei Li, Arto Mannermaa, Jaana M. Hartikainen, Javier Benitez, Anna González-Neira, Christopher A. Haiman, Thilo Dörk, Natalia V. Bogdanova, Soo Hwang Teo, Nur Aishah Mohd Taib, Olivia Fletcher, Nichola Johnson, Mervi Grip, Robert Winqvist, Carl Blomqvist, Heli Nevanlinna, Annika Lindblom, Camilla Wendt, Vessela N. Kristensen, NBCS Collaborators, Rob A. E. M. Tollenaar, Bernadette A. M. Heemskerk-Gerritsen, Paolo Radice, Bernardo Bonanni, Ute Hamann, Mehdi Manoochehri, James V. Lacey, Maria Elena Martinez, Alison M. Dunning, Paul D. P. Pharoah, Douglas F. Easton, Keun-Young Yoo, Daehee Kang

**Affiliations:** 1 Department of Biomedical Sciences, Seoul National University Graduate School, Seoul 03080, Korea; terran2712@snu.ac.kr (J.P.); chungsk@neca.re.kr (S.C.); suepark@snu.ac.kr (S.K.P.); dhkang@snu.ac.kr (D.K.); 2 BK21plus Biomedical Science Project, Seoul National University College of Medicine, Seoul 03080, Korea; 3 Institute of Health Policy and Management, Seoul National University Medical Research Center, Seoul 03080, Korea; cksjwjsja@snu.ac.kr; 4 Cancer Research Institute, Seoul National University, Seoul 03080, Korea; hanw@snu.ac.kr (W.H.); dynoh@snu.ac.kr (D.-Y.N.); 5 College of Pharmacy, Chungbuk National University, Cheongju-si 28160, Korea; nan.song@chungbuk.ac.kr; 6 Department of Preventive Medicine, Seoul National University College of Medicine, Seoul 03080, Korea; kyyoo@snu.ac.kr; 7 Department of Surgery, Seoul National University College of Medicine, Seoul 03080, Korea; 8 Department of Surgery, Medicine and ASAN Medical Center, University of Ulsan College, Seoul 05505, Korea; ahnsh@amc.seoul.kr (S.-H.A.); leejw@amc.seoul.kr (J.W.L.); 9 Division of Cancer Epidemiology and Management, National Cancer Center, Goyang-si 10408, Korea; alrud@ncc.re.kr; 10 Department of Epidemiology and Health Promotion, Institute for Health Promotion, Graduate School of Public Health, Yonsei University, Seoul 03722, Korea; jsunha@yuhs.ac; 11 Division of Epidemiology, Department of Medicine, Vanderbilt University Medical Center, Nashville, TN 37232, USA; wanqing.wen@vanderbilt.edu; 12 Centre for Cancer Genetic Epidemiology, Department of Public Health and Primary Care, University of Cambridge, Cambridge CB2 0SR, UK; mkh39@medschl.cam.ac.uk (M.K.B.); qw232@medschl.cam.ac.uk (Q.W.); jgd29@cam.ac.uk (J.D.); kyriakimi@cing.ac.cy (K.M.); pp10001@medschl.cam.ac.uk (P.D.P.P.); dfe20@medschl.cam.ac.uk (D.F.E.); 13 Biostatistics Unit, The Cyprus Institute of Neurology & Genetics, Nicosia 2371, Cyprus; 14 Cyprus School of Molecular Medicine, The Cyprus Institute of Neurology & Genetics, Nicosia 23462, Cyprus; 15 Centre for Cancer Genetic Epidemiology, Department of Oncology, University of Cambridge, Cambridge CB1 8RN, UK; ms483@medschl.cam.ac.uk (M.S.); dmc40@medschl.cam.ac.uk (D.M.C.); pah25@medschl.cam.ac.uk (P.A.H.); rm480@medschl.cam.ac.uk (R.M.); amd24@medschl.cam.ac.uk (A.M.D.); 16 Department of Medical Epidemiology and Biostatistics, Karolinska Institutet, 171 65 Stockholm, Sweden; kamila.czene@ki.se (K.C.); Per.Hall@ki.se (P.H.); 17 Department of Oncology, Södersjukhuset, 118 83 Stockholm, Sweden; 18 Department of Population Science, American Cancer Society, Atlanta, GA 30303, USA; lauren.teras@cancer.org; 19 Department of Laboratory Medicine and Pathology, Mayo Clinic, Rochester, MN 55905, USA; alpa.patel@cancer.org (A.V.P.); couch.fergus@mayo.edu (F.J.C.); 20 Department of Health Sciences Research, Mayo Clinic, Rochester, MN 55905, USA; olsonj@mayo.edu; 21 School of Cancer & Pharmaceutical Sciences, Comprehensive Cancer Centre, Guy’s Campus, King’s College London, London SE1 9RT, UK; elinor.sawyer@kcl.ac.uk; 22 Department of Oncology, UCLH Foundation Trust, London NW1 2PG, UK; r.roylance@ucl.ac.uk; 23 Copenhagen General Population Study, Herlev and Gentofte Hospital, Copenhagen University Hospital, 2730 Herlev, Denmark; stig.egil.bojesen@regionh.dk; 24 Department of Clinical Biochemistry, Herlev and Gentofte Hospital, Copenhagen University Hospital, 2730 Herlev, Denmark; 25 Faculty of Health and Medical Sciences, University of Copenhagen, 2200 Copenhagen, Denmark; 26 Department of Breast Surgery, Herlev and Gentofte Hospital, Copenhagen University Hospital, 2730 Herlev, Denmark; henrik.flyger@regionh.dk; 27 VIB Center for Cancer Biology, 3001 Leuve, Belgium; diether.lambrechts@kuleuven.vib.be; 28 Laboratory for Translational Genetics, Department of Human Genetics, University of Leuven, 3000 Leuven, Belgium; 29 Department of Radiotherapy Oncology, KU Leuven—University of Leuven, University Hospitals Leuven, 3000 Leuven, Belgium; Adinda.Baten@uzleuven.be; 30 Division of Cancer Epidemiology and Prevention, Aichi Cancer Center Research Institute, Nagoya 464-8681, Japan; kmatsuo@aichi-cc.jp; 31 Division of Cancer Epidemiology, Nagoya University Graduate School of Medicine, Nagoya 466-8550, Japan; hidemi@aichi-cc.jp; 32 Center for Research in Epidemiology and Population Health (CESP), Team Exposome and Heredity, INSERM, University Paris-Saclay, 94805 Villejuif, France; pascal.guenel@inserm.fr (P.G.); therese.truong@inserm.fr (T.T.); 33 Division of Molecular Pathology, The Netherlands Cancer Institute—Antoni van Leeuwenhoek Hospital, 1066 CX Amsterdam, The Netherlands; r.keeman@nki.nl (R.K.); mk.schmidt@nki.nl (M.K.S.); 34 Division of Psychosocial Research and Epidemiology, The Netherlands Cancer Institute—Antoni van Leeuwenhoek Hospital, 1066 CX Amsterdam, The Netherlands; 35 Department of Preventive Medicine, Keck School of Medicine, University of Southern California, Los Angeles, CA 90033, USA; Anna.Wu@med.usc.edu (A.H.W.); Chiu-Chen.Tseng@med.usc.edu (C.-C.T.); Christopher.Haiman@med.usc.edu (C.A.H.); 36 Sheffield Institute for Nucleic Acids (SInFoNiA), Department of Oncology and Metabolism, University of Sheffield, Sheffield S10 2TN, UK; a.cox@shef.ac.uk; 37 Academic Unit of Pathology, Department of Neuroscience, University of Sheffield, Sheffield S10 2TN, UK; s.s.cross@sheffield.ac.uk; 38 Peter MacCallum Cancer Center, Melbourne, VIC 3000, Australia; heather.thorne@petermac.org; 39 Sir Peter MacCallum Department of Oncology, The University of Melbourne, Melbourne, VIC 3000, Australia; 40 Fred A, Litwin Center for Cancer Genetics, Lunenfeld-Tanenbaum Research Institute of Mount Sinai Hospital, Toronto, ON M5G 1X5, Canada; andrulis@lunenfeld.ca; 41 Department of Molecular Genetics, University of Toronto, Toronto, ON M5S 1A8, Canada; 42 Centre for Epidemiology and Biostatistics, Melbourne School of Population and Global Health, The University of Melbourne, Melbourne, VIC 3010, Australia; j.hopper@unimelb.edu.au; 43 Precision Medicine, School of Clinical Sciences at Monash Health, Monash University, Clayton, VIC 3168, Australia; melissa.southey@monash.edu; 44 Department of Clinical Pathology, The University of Melbourne, Melbourne, VIC 3010, Australia; 45 Cancer Epidemiology Division, Cancer Council Victoria, Melbourne, VIC 3004, Australia; 46 Taiwan Biobank, Institute of Biomedical Sciences, Academia Sinica, Taipei 115, Taiwan; peiei@gate.sinica.edu.tw; 47 Institute of Biomedical Sciences, Academia Sinica, Taipei 115, Taiwan; bmcys@ibms.sinica.edu.tw; 48 School of Public Health, China Medical University, Taichung 404, Taiwan; 49 Department of Medicine Division of Hematology and Oncology, David Geffen School of Medicine, University of California, Los Angeles, CA 90095, USA; peter.fasching@fau.de; 50 Department of Gynecology and Obstetrics, Comprehensive Cancer Center ER-EMN, University Hospital Erlangen, Friedrich-Alexander-University Erlangen-Nuremberg, 91054 Erlangen, Germany; 51 Institute of Human Genetics, Comprehensive Cancer Center Erlangen-EMN, University Hospital Erlangen, Friedrich-Alexander University Erlangen-Nuremberg, 91054 Erlangen, Germany; Arif.Ekici@uk-erlangen.de; 52 Division of Population Health, Health Services Research and Primary Care, School of Health Sciences, Faculty of Biology, Medicine and Health, The University of Manchester, Manchester M13 9PL, UK; kenneth.muir@manchester.ac.uk (K.M.); artitaya.lophatananon@manchester.ac.uk (A.L.); 53 Division of Clinical Epidemiology and Aging Research, German Cancer Research Center (DKFZ), 69120 Heidelberg, Germany; h.brenner@dkfz.de (H.B.); v.arndt@dkfz.de (V.A.); 54 Division of Preventive Oncology, German Cancer Research Center (DKFZ) and National Center for Tumor Diseases (NCT), 69120 Heidelberg, Germany; 55 German Cancer Consortium (DKTK), German Cancer Research Center (DKFZ), 69120 Heidelberg, Germany; 56 Division of Genetics and Epidemiology, The Institute of Cancer Research, London SM2 5NG, UK; michael.jones@icr.ac.uk (M.E.J.); Anthony.Swerdlow@icr.ac.uk (A.J.S.); 57 Division of Breast Cancer Research, The Institute of Cancer Research, London SW7 3RP, UK; 58 Dr. Margarete Fischer-Bosch-Institute of Clinical Pharmacology, 70376 Stuttgart, Germany; reiner.hoppe@ikp-stuttgart.de; 59 University of Tübingen, 72074 Tübingen, Germany; 60 Department of Internal Medicine, Evangelische Kliniken Bonn gGmbH, Johanniter Krankenhaus, 53177 Bonn, Germany; Yon-Dschun.Ko@bn.johanniter-kliniken.de; 61 Saw Swee Hock School of Public Health, National University of Singapore and National University Health System, Singapore 117549, Singapore; ephbamh@nus.edu.sg; 62 Department of Surgery, Yong Loo Lin School of Medicine, National University of Singapore and National University Health System, Singapore 119228, Singapore; 63 Department of Surgery, National University Health System, Singapore 119228, Singapore; 64 Human Genetics Division, Genome Institute of Singapore, Singapore 138672, Singapore; lijm1@gis.a-star.edu.sg; 65 Translational Cancer Research Area, University of Eastern Finland, 70210 Kuopio, Finland; arto.mannermaa@uef.fi (A.M.); jaana.hartikainen@uef.fi (J.M.H.); 66 Institute of Clinical Medicine, Pathology and Forensic Medicine, University of Eastern Finland, 70210 Kuopio, Finland; 67 Biobank of Eastern Finland, Kuopio University Hospital, 70210 Kuopio, Finland; 68 Biomedical Network on Rare Diseases (CIBERER), 28029 Madrid, Spain; jbenitez@cnio.es; 69 Human Cancer Genetics Programme, Spanish National Cancer Research Centre (CNIO), 28029 Madrid, Spain; agonzalez@cnio.es; 70 Gynaecology Research Unit, Hannover Medical School, 30625 Hannover, Germany; doerk.thilo@mh-hannover.de (T.D.); bogdanova.natalia@mh-hannover.de (N.V.B.); 71 Department of Radiation Oncology, Hannover Medical School, 30625 Hannover, Germany; 72 NN Alexandrov Research Institute of Oncology and Medical Radiology, 223040 Minsk, Belarus; 73 Breast Cancer Research Programme, Cancer Research Malaysia, Subang Jaya 47500, Malaysia; soohwang.teo@cancerresearch.my; 74 Department of Surgery, Faculty of Medicine, University of Malaya, Kuala Lumpur 50603, Malaysia; 75 Breast Cancer Research Unit, University Malaya Cancer Research Institute, Faculty of Medicine, University of Malaya, Kuala Lumpur 50603, Malaysia; naisha@um.edu.my; 76 The Breast Cancer Now Toby Robins Research Centre, The Institute of Cancer Research, London SW7 3RP, UK; Olivia.fletcher@icr.ac.uk (O.F.); Nichola.johnson@icr.ac.uk (N.J.); 77 Department of Surgery, Oulu University Hospital, University of Oulu, 90220 Oulu, Finland; mervi.grip@ppshp.fi; 78 Laboratory of Cancer Genetics and Tumor Biology, Cancer and Translational Medicine Research Unit, Biocenter Oulu, University of Oulu, 90570 Oulu, Finland; robert.winqvist@oulu.fi; 79 Laboratory of Cancer Genetics and Tumor Biology, Northern Finland Laboratory Centre Oulu, Oulu 90570, Finland; 80 Department of Oncology, Helsinki University Hospital, University of Helsinki, 00290 Helsinki, Finland; carl.blomqvist@helsinki.fi; 81 Department of Oncology, Örebro University Hospital, 70185 Örebro, Sweden; 82 Department of Obstetrics and Gynecology, Helsinki University Hospital, University of Helsinki, 00290 Helsinki, Finland; Heli.Nevanlinna@hus.fi; 83 Department of Molecular Medicine and Surgery, Karolinska Institutet, 171 76 Stockholm, Sweden; Annika.Lindblom@ki.se; 84 Department of Clinical Genetics, Karolinska University Hospital, 171 76 Stockholm, Sweden; 85 Department of Clinical Science and Education, Södersjukhuset, Karolinska Institutet, 118 83 Stockholm, Sweden; camilla.wendt@sll.se; 86 Department of Medical Genetics, Oslo University Hospital and University of Oslo, 0450 Oslo, Norway; v.n.kristensen@medisin.uio.no (V.N.K.); greal@ous-hf.no (NBCS Collaborators); 87 Institute of Clinical Medicine, Faculty of Medicine, University of Oslo, 0372 Oslo, Norway; 88 Department of Research, Vestre Viken Hospital, 3004 Drammen, Norway; 89 Department of Tumor Biology, Institute for Cancer Research, Oslo University Hospital, 0450 Oslo, Norway; 90 Department of Cancer Genetics, Institute for Cancer Research, Oslo University Hospital-Radiumhospitalet, 0450 Oslo, Norway; 91 Section for Breast- and Endocrine Surgery, Department of Cancer, Division of Surgery, Cancer and Transplantation Medicine, Oslo University Hospital-Ullevål, 0450 Oslo, Norway; 92 Department of Radiology and Nuclear Medicine, Oslo University Hospital, 0450 Oslo, Norway; 93 Department of Pathology at Akershus University Hospital, 1478 Lørenskog, Norway; 94 Department of Oncology, Division of Surgery and Cancer and Transplantation Medicine, University Hospital-Radiumhospitalet, 0405 Oslo, Norway; 95 National Advisory Unit on Late Effects after Cancer Treatment, Department of Oncology, Oslo University Hospital, 0405 Oslo, Norway; 96 Department of Oncology, Akershus University Hospital, 1478 Lørenskog, Norway; 97 Oslo Breast Cancer Research Consortium, Oslo University Hospital, 0405 Oslo, Norway; 98 Department of Surgery, Leiden University Medical Center, 2333 ZA Leiden, The Netherlands; r.a.e.m.tollenaar@lumc.nl; 99 Department of Medical Oncology, Erasmus MC Cancer Institute, 3015 CN Rotterdam, The Netherlands; b.heemskerk-gerritsen@erasmusmc.nl; 100 Unit of Molecular Bases of Genetic Risk and Genetic Testing, Department of Research, Fondazione IRCCS Istituto Nazionale dei Tumori (INT), 20133 Milan, Italy; Paolo.Radice@istitutotumori.mi.it; 101 Division of Cancer Prevention and Genetics, IEO, European Institute of Oncology IRCCS, 20141 Milan, Italy; bernardo.bonanni@ieo.it; 102 Molecular Genetics of Breast Cancer, German Cancer Research Center (DKFZ), 69120 Heidelberg, Germany; u.hamann@dkfz-heidelberg.de (U.H.); mehdi.manoochehri@igb.fraunhofer.de (M.M.); 103 Department of Computational and Quantitative Medicine, City of Hope, Duarte, CA 91010, USA; jlacey@coh.org; 104 City of Hope Comprehensive Cancer Center, City of Hope, Duarte, CA 91010, USA; 105 Moores Cancer Center, University of California San Diego, La Jolla, CA 92037, USA; e8martinez@health.ucsd.edu; 106 Herbert Wertheim School of Public Health and Longevity Science, University of California San Diego, La Jolla, CA 92161, USA

**Keywords:** breast cancer, estrogen, gene-environment interaction

## Abstract

**Simple Summary:**

Breast cancer is the most common cancer in females worldwide. To date, many gene–environment interaction (GxE) studies have been conducted to better understand how genetic factors combine with environmental factors to influence risk. However, previous studies have not found or found only a few interactions by using SNPs which were discovered from genome-wide association studies and have been conducted, for the most part, within European populations. In this study, we focused on estrogen-related lifestyle factors that have been identified for breast cancer, including several well-established reproductive factors that are mediated by hormonal mechanisms. We aimed to examine whether there are any gene and environmental factor interactions related to estrogen exposure or metabolism using a candidate approach in Korean women. We found two interactions in this study, although they were not replicated in the independent large consortium data. These findings suggest specificity in Koreans for breast cancer risk.

**Abstract:**

In this study we aim to examine gene–environment interactions (GxEs) between genes involved with estrogen metabolism and environmental factors related to estrogen exposure. GxE analyses were conducted with 1970 Korean breast cancer cases and 2052 controls in the case-control study, the Seoul Breast Cancer Study (SEBCS). A total of 11,555 SNPs from the 137 candidate genes were included in the GxE analyses with eight established environmental factors. A replication test was conducted by using an independent population from the Breast Cancer Association Consortium (BCAC), with 62,485 Europeans and 9047 Asians. The GxE tests were performed by using two-step methods in GxEScan software. Two interactions were found in the SEBCS. The first interaction was shown between rs13035764 of NCOA1 and age at menarche in the GE|2df model (*p*-2df = 1.2 × 10^−3^). The age at menarche before 14 years old was associated with the high risk of breast cancer, and the risk was higher when subjects had homozygous minor allele G. The second GxE was shown between rs851998 near ESR1 and height in the GE|2df model (*p*-2df = 1.1 × 10^−4^). Height taller than 160 cm was associated with a high risk of breast cancer, and the risk increased when the minor allele was added. The findings were not replicated in the BCAC. These results would suggest specificity in Koreans for breast cancer risk.

## 1. Introduction

Breast cancer is the most common cancer in females worldwide, with an estimated 2.3 million incident cases globally in 2020. Moreover, breast cancer ranks as the most common cancer for women in 159 countries, and the most common cause of cancer deaths in 110 countries [1]. In Korea, the incidence rate of breast cancer, as well as breast cancer mortality, has been persistently increasing [2].

Numerous epidemiological studies have been performed to identify risk factors for breast cancer and many non-genetic factors, referred to as environmental factors, have been established [3,4,5]. Furthermore, many genome-wide association studies (GWAS) have been conducted, which have provided significant opportunities to discover the potential effects of common genetic factors on complex diseases [6]. To date, more than 200 common susceptibility loci for breast cancer have been identified through GWAS [7,8,9,10]. Although *BRCA1* and *BRCA2* are the most well-known risk genes associated with about a 20-fold increased breast cancer risk, they only account for 20% of familial breast cancer due to the low frequency of mutations [11,12]. Even if all the common genetic factors are taken together, they are estimated to explain only about 30% of the familial risk [7]. Consequently, further research is needed to identify the missing heritability [13], and to better understand how genetic factors combine with environmental factors to influence risk [14].

Currently, a few alternatives have been proposed, and one of them is a gene-environment interaction (GxE) analysis. By accounting for interactions between genetic and environmental factors, better estimates of the population-attributable risk or effects in specific subgroups exposed by certain environments can be obtained [15,16]. To date, many GxE studies for breast cancer risk have been conducted, but few studies have shown significant results after accounting for multiple testing [14,17,18,19,20,21,22,23,24]. Most of these studies used the standard case-control analysis of GxE interaction based on logistic regression and added interaction terms and tended to have poor statistical power. Some methods have been developed and proposed [25], and two-step approaches have been reported to have greater power than other approaches [13]. However, previous studies have not found, or have only found a few interactions by using SNPs which were discovered from GWAS and have been conducted, for the most part, within European populations [14,17,18,19,20,21,22,23,24].

In this study we focused on estrogen-related lifestyle factors that have been identified for breast cancer, including several well-established reproductive factors that are mediated by hormonal mechanisms. Endogenous estrogen levels are likely controlled by genetic factors of the estrogen metabolism pathway and, in turn, play an integral part as determinants of breast cancer risk [26,27,28]. Thus, we aimed to examine whether there are any interactions between genes and environmental factors related to estrogen exposure or metabolism by using a candidate approach in Korean women.

## 2. Results

Table 1 shows the distribution of demographic and reproductive factors and their association with breast cancer risk in the Seoul Breast Cancer Study (SEBCS) population. Controls were generally older than cases, and most of the environmental factors were significantly different between cases and controls. Taller height, lower BMI, younger age at menarche, older age at first full term pregnancy (FFTP), no breastfeeding experience and shorter duration of breastfeeding, and longer duration of estrogen exposure before FFTP (EEBF) were associated with increased risk for breast cancer among the eight candidate environmental factors.

We conducted the replication using both a European population and an Asian population in the Breast Cancer Association Consortium (BCAC) (Table S3), and their distribution of demographic and other factors are shown in Tables S4 and S5, as well as associations with breast cancer. The associations with the risk of breast cancer were heterogeneous by ethnicity in height, age at menarche, age at FFTP, and menopause status.

We found two interactions through the GxE analysis (Table 2). High LDs were observed among the three SNPs (i.e., rs13035764, rs11125629, and rs11688818) in NCOA1 on chromosome 2.

These three SNPs, i.e., rs13035764, rs11125629, and rs11688818, showed interactions with age at menarche by the weighted GE|2df method (p_2df_ = 0.0012, 0.0018, 0.0027 respectively, significant threshold *p* = 0.005, bin1) (Figure S1). Age at menarche before 14 years old (<25% quartile) was associated with increased risk of breast cancer (OR = 1.27, 95% CI 1.07–1.50); this association was much stronger for subjects who had a homozygous minor allele G (OR_age at menarche < 14 years|rs13035764_GG_ = 2.85, 95% CI 1.43–5.67). When we examined the associations of age at menarche with breast cancer risk in 44 studies, eight studies (seven European and one Asian) showed consistent effects with the SEBCS (Figure S3), however, interaction with rs13035764 was not observed in the replication set from either theEuropean or Asian populations (Table 3 and Table S6).

Figure S2 shows the results of the interaction between height and three SNPs (rs851998, rs851971, and rs851967) located near the ESR1 gene in chromosome 6 by the subset GE|2df method (p_2df_ = 0.000068, 0.000094 and 0.0001, respectively, the significance threshold was 0.000110 because the number of SNPs tested in step 2 was 456). Height taller than 160 cm (≥75% quartile) was associated with increased risk of breast cancer (OR = 1.92, 95% CI 1.67–2.20). The association of height with breast cancer was stronger when the minor allele was considered (OR_height ≥ 160 cm|rs851998_GG_ = 1.53, 95% CI 1.20–1.95; OR_height ≥ 160 cm|rs851998_GA_ = 2.03, 95% CI 1.65–2.49; and OR_height ≥ 160 cm|rs851998_AA_ = 2.39, 95% CI 1.74–3.29) (Table 3). This association was observed among Asian studies of the BCAC. The risk of breast cancer associated with greater height was enhanced among those who had minor alleles (OR_height ≥ 160 cm|rs851998_AA_ = 1.38, 95% CI 1.04–1.82) but the interaction test was not significant (*p*-GxE = 0.4642) (Table 3). Although there were ten studies (nine European and one Asian) that showed the consistent effects of height with SEBCS (Figure S4), a significant interaction with rs851998 was not found, even in the combined population, which showed a consistent effect with the SEBCS (Table S7).

The associations between each environmental factor and the risk of breast cancer by genotype were not different according to the estrogen receptor (ER) or progesterone receptor (PR) status in the SEBCS population (Table S8).

## 3. Discussion

We performed GxE analyses between 11,555 SNPs in and near the promoters of the 136 genes involved with estrogen metabolism and eight environmental factors related to estrogen exposure by using GxEScan. Various results from the two-step methods were obtained, and two interactions involving NCOA1 with age at menarche and ESR1 with height were found.

The minor allele of rs13035764 in the NCOA1 was associated with an enhanced risk of breast cancer for younger age at menarche in the SEBCS. Our study suggested that women whose menstruation was late, possibly had an increased protective effect with this variant. NCOA1 is known as the steroid receptor coactivator 1 (SRC-1) encoding a protein that acts as a transcriptional coactivator for steroid and nuclear hormone receptors. Including SRC-1, SRC coactivators have important roles in development, growth, reproduction, and even in cancer [29]. It has been shown that SRC-1 proteins were overexpressed from 19% to 29% of human breast cancers, and this overexpression has been associated with large and high-grade tumors [30,31,32]. Although there was no epidemiological study that examined the association between SRC-1 and age at menarche directly, previous reports have indicated the regulatory role of SRC-1 during a specific phase in the menstrual cycle [33,34]. Thus, our findings could suggest that the risk for breast cancer decreases in the late menarche group as they were exposed not only to the hormone but also to the SRC-1 effects later.

We also found that the minor allele of rs851998, which is located near the ESR1 gene, was associated with an increased risk of breast cancer for females who were taller than 160 cm. Previously, numerous studies have suggested the association between the ESR1 gene and breast cancer [35,36,37,38], and have reported the role of ESR1 mutations for breast cancer [39,40,41]. In addition, adult height has been linked with breast cancer risk from many epidemiological studies [42,43,44,45,46]. As a key factor for the development of female sex organs and sex characteristics, estrogen also plays a role in height as a part of the growth process [47,48]. Although which part of estrogen metabolism and how it acts on the growth in vivo is unclear [49], many studies have supported that because estrogen synthesis and secretion are increased during puberty, these factors could lead to epiphyseal fusion, termination of linear growth, and determination of final height [50,51,52,53]. With several reports suggesting the association between ESR1 and height [54,55,56], we think that the ESR1 gene and height possibly have an association with breast cancer risk, since significant interaction results were found in our results.

This study was conducted by the candidate gene approach with a hypothesis for statistical efficiency and biological plausibility. There have been a considerable number of criticisms [57,58,59]. The main concerns have included inadequate analytic procedures during the selection of genetic or environmental factors and using statistical methods with small sample size and lower power, as well as publication bias. However, an opposite commentary has refuted that the candidate GxE approach remains to be the most commonly studied despite its limitations, and the goal of a candidate gene-environment interaction study is distinct from that of a GWAS-based interaction study [60]. GxE based on GWAS focuses on gene-environmental correlations, while candidate GxE with a hypothesis attempts to discover causes. Moreover, GxE based on GWAS mainly aims to overcome missing heritability and to examine the modified effect of genetic factors by environmental factors. There are other approaches to discover novel genetic factors, which have interactions with established environmental factors from genome-wide ranges; however, these approaches could not report significant interactions, possibly due to the burden of multiple testing [20,21]. Meanwhile, the candidate GxE approach, which aimed to detect genetic factors that have interactions with established environmental factors from candidate genes and to examine the modified effects of environmental factors by genotype, could decrease the statistical burden and interpret easily due to biological plausibility.

There are several limitations to this study. First, we dichotomized the continuous variables when the environmental factors were included in the GxE analysis. Although this approach could have some statistical problems, such as losing information, underestimating the extent of variation, and concealing nonlinearity, binary variables make interpretation easy with possible mechanistic conclusions [61] and facilitate testing for multi-factor interactions [62]. Second, our statistically significant results were obtained from the EG|2df method rather than from the EDGxE, which demonstrated the best power and efficiency. Furthermore, the 2df test is called the joint gene and GxE test, in which the hypothesis is βg = βgxe = 0. Thus, a polymorphism can be detected even when there is no interaction, but it has a marginal effect. However, our results from the GE|2df showed that the effect of environmental factors differed according to the number of minor alleles (Table 3). Thus, they might not be false-positive results. Third, the interactions found from the SEBCS were not replicated in the independent large consortium data, even in the replication test using each ethnicity population and combined population which showed consistent effects with the SEBCS. To date, numerous studies have performed gene-environment interaction tests for breast cancer risk, however, the results have rarely been replicated [63,64,65]. Most of the studies have been conducted in the European population and all replicated results have also been found in the European descent study population. This could be explained by racial differences impacting environmental factors and also genetic backgrounds [66,67,68,69,70] (as shown the Figures S3 and S4 and Tables S6 and S7). A larger study population or consortium data including Asian descent are demanded to replicate the findings successfully. Fourth, the sample size of this study was not large relative to other consortium studies; however, two-step methods generally require a smaller sample size to achieve enough power for the GxE test as compared with the standard GxE test, and a previous study used only 2382 subjects and found novel genes by using GxEScan [13]. Lastly, breast cancer was considered as a whole entity rather than subtypes of breast cancers such as pre-menopausal and post-menopausal breast cancer due to the limitation of sample size. Further studies with a larger sample size would find that differences in the GxE depend on menopause status.

Although breast cancer is the most common cancer in women worldwide, the incidence or mortality of breast cancer differs from country to country [71]. According to GLOBCAN 2018 and the Korean Breast Cancer Society, the incidence rate of most of these countries is decreasing, while it is still increasing in Korea. The mortality of breast cancer is also dramatically increasing [72,73]. Korean females have shown a decreasing trend of age at menarche and an increasing trend of height [74,75,76,77]. These aspects possibly explain why the incidence rate of breast cancer is increasing in Korea, and therefore there is a need for research on breast cancer specific to Korean women.

## 4. Methods

### 4.1. Study Population of the Gene-Environment Interaction (GxE) Study

The Seoul Breast Cancer Study (SEBCS) subjects were used in this study. As a multicenter case-control study, 4040 women with histologically confirmed breast cancer were recruited between 2001 and 2007 from the Seoul National University Hospital and the Asan Medical Center. During the same period, 1818 non-cancer patients aged 32 to 75 years were enrolled as hospital-based controls in the same hospital, and 2052 healthy women aged 39 to 71 years who were participants in a community health screening program from 2002 to 2007 were also included in the control group [78].

A questionnaire survey was administered by trained interviewers using in-person interviews for all subjects. Demographic information, family history, behavioral and dietary habits, and reproductive information were collected by this survey. Among the participants, we excluded those who had previous histories of cancer, hysterectomy, or oophorectomy, and only included those who were born from 1930 to 1969, following the same criteria as a previous study [78]. From these exclusion criteria, 3332 cases and 3620 controls were selected with questionnaire data. All subjects in the SEBCS had written informed consent, and the study design was approved by the Committee on Human Research of the Seoul National University Hospital (IRB no. H-0503-144-004).

### 4.2. Study Population and Data Used in the Replication

A large dataset pooled from the Breast Cancer Association Consortium (BCAC) was used for the replication. Participants who were not of European or Asian descent and had missing data regarding sex, age, and genotype information of SNPs were excluded. A replication dataset for interaction between age at menarche and an SNP that had shown interaction with age at menarche from the SEBCS was made after excluding subjects who had missing data on age at menarche. Likewise, a replication dataset for interaction between height and an SNP that had shown an interaction with height from the SEBCS was created after excluding missing data in height. Missing values of covariates including family history, age at first full term pregnancy, and menopause status were categorized separately. In total, 62,485 women of European descent (36,894 cases and 25,591 controls in 37 studies) and 9047 women of Asian descent (4392 cases and 4655 controls in 7 studies) were included in the replication dataset to examine the interaction between age at menarche and an SNP in NCOA1, and 56,363 women of European descent (31,994 cases and 24,369 controls in 29 studies) and 5789 women of Asian descent (2879 cases and 2910 controls in 4 studies) were included in a replication dataset to assess the interaction between height and an SNP in ESR1 (Table S3).

### 4.3. GWAS Data

The SEBCS also performed GWAS by using the Affymetrix Genome-Wide Human SNP Array 6.0 chip (Affymetrix, Inc., Santa Clara, CA, USA). A total of 4394 women (2342 cases and 2052 controls) were scanned, and we compiled a final dataset that included 555,117 SNPs after quality control exclusions. Detailed descriptions of genotyping and quality control procedures can be found in the previous literature [79,80]. To conduct gene-environment interaction analyses, we included as eligible subjects a total 4022 women (1970 cases and 2052 controls) who had both questionnaire and GWAS data.

### 4.4. Candidate Gene Selection

We used the search term “estrogen metabolism” in several databases to identify candidate genes involved with estrogen metabolism. Ninety-one genes were identified using the Ensembl genome browser 84 (www.ensembl.org/ (accessed on 30 May 2016)), and 89 genes were selected except for two genes located on the X chromosome. Twenty-eight genes were searched using AmiGO (http://amigo1.geneontology.org/ (accessed on 30 May 2016)), but all genes overlapped with the previous selection. Twenty-nine genes were searched using AmiGO2 (http://amigo.geneontology.org/amigo (accessed on 30 May 2016)), and two genes, which did not overlap with the previous selection, were added. We also searched the literature to find more genes related to estrogen metabolism. Thirty-seven genes were added among 199 SNPs, which showed significant results from the Kyoto Encyclopedia of Genes and Genomes (KEGG) pathway analysis [81], and nine genes were further added from previous studies [82,83,84,85,86,87,88,89,90,91,92,93,94]. A total of 137 candidate genes were on our list.

### 4.5. SNPs Extraction and Imputation

The location and range of candidate genes data were searched using Ensembl genome browser 84 and the National Center for Biotechnology Information (NCBI) website (GRCh38/hg38 ver.). To cover the promoter regions of most candidate genes, we included SNPs from candidate genes that were within 20 Kb from the start and the end positions of each range [95]. Of the 555,117 SNPs mentioned in the above GWAS data, 2482 SNPs were extracted from 136 genes and one gene had an empty extraction result. After the Hardy–Weinberg equilibrium (HWE) test, 2472 SNPs were selected with HWE > 0.001.

We also performed an SNP imputation based on HapMap 2.0 (release 22/hg18) with the same range of candidate genes. In total, 9750 SNPs with quality scores > 0.3 were obtained by imputation, and 9083 SNPs with minor allele frequency (MAF) > 0.01 were included in the GxE analysis. In total, 11,555 SNPs were tested in the GxE analysis.

### 4.6. Environmental Factors Related to Estrogen Exposure

We selected seven variables related to estrogen exposure in this analysis including body mass index (BMI), height, age at menarche, age at first full term pregnancy (FFTP), number of children, breastfeeding, and total duration of breastfeeding. All exposures were continuous variables, except for breastfeeding which was collected as yes/no, and most of the continuous variables had missing values. We performed an imputation for missing data to the median value of each variable in the stratified birth year groups (1930–1950, 1950–1959, and 1960–1969), and therefore the effect of birth cohort could be considered without reducing power with the sample size (Table 1 and Table S1).

To assess the adjusted effect of breastfeeding duration, breastfeeding duration per child was calculated by dividing the total duration of breastfeeding by the number of children. We also made a new variable to examine the combined effects of age at menarche and age at FFTP. The duration of estrogen exposure before FFTP (EEBF) was calculated by subtracting the age at menarche from the age at FFTP for parous women. For nulliparous women, EEBF was calculated by subtracting the age at menarche from the age at interview for pre-menopause women, or from the age at menopause for post-menopause women.

To reduce computational burden and to facilitate interpretation, all continuous variables were dichotomized by quartile of risk direction. This resulted in eight environmental factors in the GxE analysis that were categorized as follows: age at menarche (≥14 years vs. <14 years, 25% quartile); age at FFTP (<27 years vs. nulliparity or ≥27 years, 75% quartile); height (<160 cm vs. ≥160 cm. 75% quartile); BMI (<25 kg/m^2^ vs. ≥25 kg/m^2^ Korea criteria of obesity); number of children (≥2 vs. <2, 25% quartile); breastfeeding (often vs. never); breastfeeding duration per child (≥2.5 month vs. <2.5 month, 25% quartile); and EEBF (<13 years vs. ≥13 years, 75% quartile). Likewise, continuous variables from the BCAC were dichotomized by quartile of risk direction in the European and Asian populations, respectively, as follows: age at menarche (≥12 years vs. <12 years, 25% quartile in both European and Asian populations); height (<169 cm vs. ≥169 cm, 75% quartile in European population and <160 cm vs. ≥160 cm, 75% quartile in Asian population); and age at FFTP (<28 years vs. nulliparity or ≥28 years, 75% quartile in both European and Asian populations).

The estrogen receptor (ER) or progesterone receptor (PR) status was collected by the medical and pathological records and was determined when the tumor cells showed 10% or more positive nuclear staining from an immunohistochemistry assay [78].

### 4.7. Statistical Analysis

To estimate the effect of each environmental factor for breast cancer with odds ratios (OR) and 95% confidence intervals (CI), age, family history, parity, age at FFTP, age at menarche, and menopausal status were commonly included in the model. For variables regarding parity such as the number of children, breastfeeding history, and breastfeeding duration per child, further adjustments were performed by adding the number of children to the model. As an exception, age, family history, parity, and menopausal status were included in the model when the analysis was performed for EEBF. These models were consistently used in the GxE analysis, and in the logistic regression to estimate genetic risk factors, which showed significant interactions in the GxE analysis.

GxEScan (ver. Beta 0.4.0; http://biostats.usc.edu/software (accessed on 1 June 2016)) was mainly used in this study to conduct the GxE analysis. This software was developed by Gauderman et al. [13] and was designed to conduct GxE analysis in case-control study designs using the traditional GxE test and also a few two-step methods. Especially, the two-step methods have generally been proposed to provide greater statistical power than the traditional case-control GxE test while preserving the type I error rate. Four two-step methods were provided by GxEScan, i.e., DG|GxE, GE|GxE, EDGxE, and GE|2df and we mainly observed GxE results from these methods. There were two hypotheses testing approaches in the two-step methods, ‘subset testing’ and ‘weighted testing’. Detailed descriptions of these hypotheses can be found elsewhere [13,96] and both were used in this study.

As an initial step, we carried out GxE analyses by birth year groups (1930–1950, 1950–1959, and 1960–1969) because each birth year group had a different distribution of environmental factors (Table S2). The results of GxE analyses of each birth year group were inconsistent and were not statistically significant, possibly due to the modest sample size. For these reasons, we performed a meta-analysis of the three sets of results obtained from each birth year group by using METAL software [97], and then compared our results with the GxE result from the overall pooled data. There was a negligible difference between the two results; we presented the result from the pooled data analysis.

PLINK (ver. 1.07; http://pngu.mgh.harvard.edu/purcell/plink/ (accessed on 7 March 2016)) was used to examine GWAS data and for extracting candidate SNPs. Overall management of questionnaire data and statistical analyses were performed by SAS (ver. 9.4, SAS Institute Inc., Cary, NC, USA).

Replication analyses were performed in the two datasets for gene x height and for gene x age at menarche, separately. Interactions were tested by ethnicity first, and then in each study that showed the consistent effects of exposures with SEBCS. SAS (ver. 9.4; SAS Institute Inc., Cary, NC, USA) was used to test replication.

## 5. Conclusions

Through the GxE test between 136 candidate genes involved in estrogen metabolism and eight environmental factors related to estrogen exposure, we found two interactions in middle-aged Korean females. Although the results of our study were not replicated, they do suggest specificity in Koreans for breast cancer risk. The explainable interaction might be a better method to interpret and apply the results than deep medicine or artificial intelligence [98]. We expect to make a step forward in predicting and preventing breast cancer, as more interactions are to be investigated.

## Figures and Tables

**Table 1:** Distributions of demographic and reproductive factors and associations with the risk of breast cancer, Seoul Breast Cancer Study.

Demographic and Reproductive Factors	Cases *N* = 1970		Controls *N* = 2052		OR ^c^	95% CI
	Mean ± SD	%	Mean ± SD	%		
Age	49.0 ± 8.26		51.4 ± 7.75		0.99	0.98–1.01
Family history. yes		4.8		1.8	2.64	1.77–3.94
Height	157.9 ± 5.04		155.9 ± 5.00		1.07	1.06–1.08
<160 cm		59.6		75.7	1.00	reference
≥160 cm		40.4		24.3	1.92	1.67–2.20
BMI (kg/m^2^)	23.2 ± 2.94		23.7 ± 2.96		0.97	0.95–1.00
<25		76.8		71.2	1.00	reference
≥25		23.2		28.9	0.81	0.69–0.93
Menarche age	14.8 ± 1.65		15.2 ± 1.77		0.90	0.86–0.93
≥14 years		78.9		85.0	1.00	reference
<14 years		21.1		15.0	1.27	1.07–1.50
Ever pregnancy		93.5		92.8	0.26	0.14–0.46
Age at FFTP ^a^	25.9 ±3.48		25.1 ± 3.36		1.05	1.03–1.07
<27 years		55.6		66.4	1.00	reference
Nulliparity or ≥27 years		44.4		33.6	1.49	1.30–1.70
No. of children	2.2 ± 0.85		2.3 ± 0.91		1.06	0.97–1.16
≥2		85.2		88.2	1.00	reference
<2		14.8		11.8	1.11	0.91–1.36
Never breastfeeding		22.3		14.5	1.31 ^d^	1.10–1.57
Breastfeeding duration (months)	18.0 ± 22.59		24.0 ± 21.53		0.99 ^d^	0.99–1.00
Breastfeeding duration per child (months)	7.3 ± 8.01		9.9 ± 7.27		0.97 ^d^	0.96–0.98
≥2.5 month		45.1		68.3	1.00	reference
<2.5 month		54.9		31.7	3.81 ^d^	3.16–4.59
Duration of EEBF ^b^	12.5 ± 6.78		11.7 ± 8.00		1.04 ^e^	1.02–1.05
<13 years		61.1		76.2	1.00	reference
≥13 years		38.9		23.8	1.75 ^e^	1.51–2.03
Postmenopausal women		38.2		54.8	0.60	0.50–0.72
Age at menopause	48.5 ± 5.40		49.2 ± 4.55		0.96	0.94–0.98

^a^ FFTP first full-term pregnancy (years); ^b^ EEBF estrogen exposure before the first full-term pregnancy (years); ^c^ ORs analyzed by logistic regression adjusting for age, family history, categorical FFTP, age at menarche, and menopausal status; ^d^ ORs analyzed by logistic regression adjusting for age, family history, categorical FFTP, age at menarche, menopausal status, and the number of children; ^e^ ORs analyzed by logistic regression adjusting for age, family history, ever pregnancy, and menopausal status.

**Table 2:** Top two SNPs from the gene and environment interaction analysis between 11,555 SNPs involved in estrogen metabolism and 8 environmental factors related to estrogen, Seoul Breast Cancer Study.

**SNP**	**Chromosome**	**Position_b36**	**Gene**	**Minor/Major_Allele**	**MAF**		
rs13035764	2	24571432	*NCOA1*	G/C	0.2971		
	**N(Cases/Controls)**	**OR ^a^ (95%CI)**	**Analysis methods**	**Environment**	***p*-2df**	**Threshold**	***p*-GxE**
	3925(1949/1976)	0.84 (0.76–0.93)	Weighted GE|2df	Age at menarche	1.2 × 10^−3^	0.005 ^b^	0.0524
**SNP**	**Chromosome**	**Position_b36**	**Gene**	**Minor/Major_Allele**	**MAF**		
rs851998	6	152025031	*ESR1*	A/G	0.4426		
	**N(Cases/Controls)**	**OR ^a^ (95%CI)**	**Analysis methods**	**Environment**	***p*-2df**	**Threshold**	***p*-GxE**
	3940(1955/1985)	0.82 (0.75–0.90)	Subset GE|2df	height	6.8 × 10^−5^	0.000110 ^c^	0.0471

^a^ risk of SNP for breast cancer; ^b^ threshold of bin 1 in the weighted model; ^c^ 0.05 divided by 456 SNPs which were tested in step 2.

**Table 3:** Association between an environmental factor and breast cancer by genotypes.

Study	Exposure	Category	N(Cases/Controls)	OR	95% CI	*p*-GxE
SEBCS	Age at menarche < 14 years old ^a^	Effect of E in overall	4022(1970/2052)	1.27	1.07–1.50	0.0524
		E|rs13035764_CC	1963(1007/956)	1.19	0.95–1.51	
		E|rs13035764_CG	1706(810/896)	1.17	0.89–1.53	
		E|rs13035764_GG	337(147/190)	2.85	1.43–5.67	
	Height ≥ 160 cm ^b^	Effect of E in overall	4022(1970/2052)	1.92	1.67–2.20	0.0471
		E|rs851998_GG	1281(677/604)	1.53	1.20–1.95	
		E|rs851998_GA	1921(938/983)	2.03	1.65–2.49	
		E|rs851998_AA	819(355/464)	2.39	1.74–3.29	
BCAC-European	Age at menarche < 12 years old ^a^	Effect of E in overall	62,485(36,894/25,591)	1.03	0.99–1.08	0.5314
		E|rs13035764_CC	9953(5921/4032)	1.02	0.91–1.14	
		E|rs13035764_CG	34,124(20,140/13,984)	1.03	0.97–1.09	
		E|rs13035764_GG	18,408(10,833/7575)	1.05	0.97–1.14	
	Height ≥ 169 cm ^b^	Effect of E in overall	56,363(31,994/24,369)	0.97	0.93–1.01	0.2425
		E|rs851998_GG	27,224(15,513/11,711)	0.93	0.88–0.99	
		E|rs851998_GA	23,843(13,459/10,384)	0.99	0.93–1.05	
		E|rs851998_AA	5296(3022/2274)	1.03	0.90–1.17	
BCAC-Asian	Age at menarche < 12 years old ^a^	Effect of E in overall	9047(4392/4655)	0.97	0.82–1.14	0.7817
		E|rs13035764_CC	4867(2407/2460)	0.95	0.75–1.21	
		E|rs13035764_CG	3504(1666/1838)	0.99	0.76–1.28	
		E|rs13035764_GG	676(319/357)	1.04	0.57–1.91	
	Height ≥ 160 cm ^b^	Effect of E in overall	5789(2879/2910)	1.22	1.08–1.39	0.4642
		E|rs851998_GG	1751(899/852)	1.20	0.95–1.52	
		E|rs851998_GA	2846(1422/1424)	1.19	0.99–1.42	
		E|rs851998_AA	1192(558/634)	1.38	1.04–1.82	

ORs analyzed by logistic regression adjusting for age, family history, categorical FFTP, age at menarche, and menopausal status. ^a^ age at menarche <25% quartile; ^b^ height ≥75% quartile.

## Data Availability

Data are available on request due to restraints imposed by the ethics committees of individual. To request the data from the Breast Cancer Association Consortium (BCAC), readers are instructed to submit a concept proposal, which will be reviewed by the BCAC Data Access Coordination Committee (DACC).

## Supplementary Materials

The following are available online at https://www.mdpi.com/2072-6694/13/10/2370/s1, Figure S1: Result plot of GxE between 11,555 SNPs and age at menarche for breast cancer risk by using the weighted GE|2df method, Figure S2: Result plot of GxE between 11,555 SNPs and height for breast cancer risk by using the GE|2df method, Figure S3: Forest plot presenting risk of age at menarche for breast cancer by study in BCAC, Figure S4: Forest plot presenting risk of height for breast cancer by study in BCAC, Table S1: Distributions of demographic and reproductive factors and associations with the risk of breast cancer, Seoul Breast Cancer Study (pre-imputation), Table S2: Distributions of demographic and reproductive factors in cases and controls by birth year groups, Seoul Breast Cancer Study (imputed + binary), Table S3: Studies including BCAC replication data set, Table S4: Distributions of demographic and reproductive factors and associations with the risk of breast cancer by ethnicity, Breast Cancer Association Consortium replication dataset for age at menarche x NCOA1, Table S5: Distributions of demographic and reproductive factors and associations with the risk of breast cancer by ethnicity, Breast Cancer Association Consortium replication dataset for height x ESR1, Table S6: Association between age at menarche and the risk of breast cancer in studies from BCAC that showed consistence with SeBCS, Table S7: Association between height and the risk of breast cancer in studies from BCAC that showed consistence with SeBCS, Table S8: Association between environmental factor and breast cancer by genotypes according to ER/PR status, Seoul Breast Cancer Study.
