# Supplementary material for: Gene-Environment Interactions Relevant to Estrogen and Risk of Breast Cancer: Can Gene-Environment Interactions Be Detected Only among Candidate SNPs from Genome-Wide Association Studies?
Source: Cancers (Basel). 2021 May 14;13(10):2370. doi: 10.3390/cancers13102370 (PMC8156547; doi:10.3390/cancers13102370)
Supplement: Supplementary file 1 [file cancers-13-02370-s001.zip › cancers-1197487-supplementary.pdf]

Supplementary Materials

Gene–Environment Interactions Relevant to Estrogen and Risk of Breast Cancer: Can Gene–Environment Interactions Be Detected Only Among Candidate SNPs from Genome-Wide Association Studies?

JooYong Park, Ji-Yeob Choi, Jaesung Choi, Seokang Chung, Nan Song, Sue K. Park, Wonshik Han, Dong-Young Noh, Sei-Hyun Ahn, Jong Won Lee, Mi Kyung Kim, Sun Ha Jee, Wanqing Wen, Manjeet K. Bolla, Qin Wang, Joe Dennis, Kyriaki Michailidou, Mitul Shah, Don M. Conroy, Patricia A. Harrington, Rebecca Mayes, Kamila Czene, Per Hall, Lauren R. Teras, Alpa V. Patel, Fergus J. Couch, Janet E. Olson, Elinor J. Sawyer, Rebecca Roylance, Stig E. Bojesen, Henrik Flyger, Diether Lambrechts, Adinda Baten, Keitaro Matsuo, Hidemi Ito, Pascal Guénel, Thérèse Truong, Renske Keeman, Marjanka K. Schmidt, Anna H. Wu, Chiu-Chen Tseng, Angela Cox, Simon S. Cross, kConFab Investigators, Irene L. Andrulis, John L. Hopper, Melissa C. Southey, Pei-Ei Wu, Chen-Yang Shen, Peter A. Fasching, Arif B. Ekici, Kenneth Muir, Artitaya Lophatananon, Hermann Brenner, Volker Arndt, Michael E. Jones, Anthony J. Swerdlow, Reiner Hoppe, Yon-Dschun Ko, Mikael Hartman, Jingmei Li, Arto Mannermaa, Jaana M. Hartikainen, Javier Benitez, Anna González-Neira, Christopher A. Haiman, Thilo Dörk, Natalia V. Bogdanova, Soo Hwang Teo, Nur Aishah Mohd Taib, Olivia Fletcher, Nichola Johnson, Mervi Grip, Robert Winqvist, Carl Blomqvist, Heli Nevanlinna, Annika Lindblom, Camilla Wendt, Vessela N. Kristensen, NBCS Collaborators, Rob A.E.M. Tollenaar, Bernadette A.M. Heemskerk-Gerritsen, Paolo Radice, Bernardo Bonanni, Ute Hamann, Mehdi Manoochehri, James V. Lacey, Maria Elena Martinez, Alison M. Dunning, Paul D.P. Pharoah, Douglas F. Easton, Keun-Young Yoo and Daehee Kang

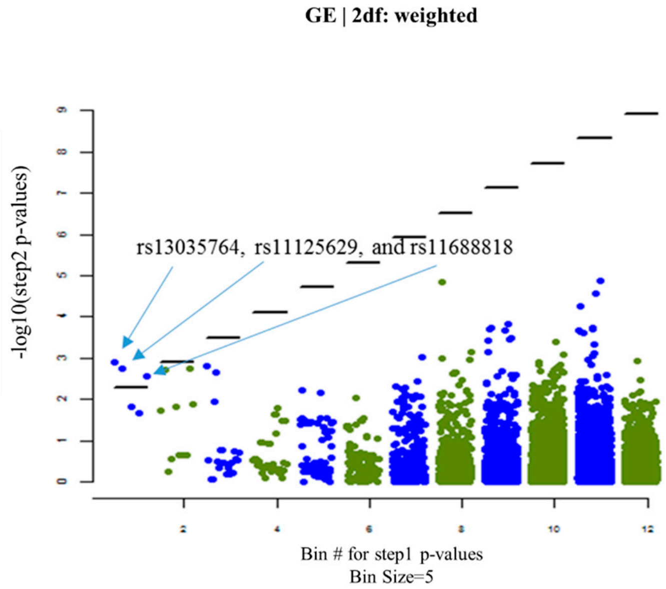

Figure S1. Result plot of GxE between 11,555 SNPs and age at menarche for breast cancer risk by using weighted GE|2df method.

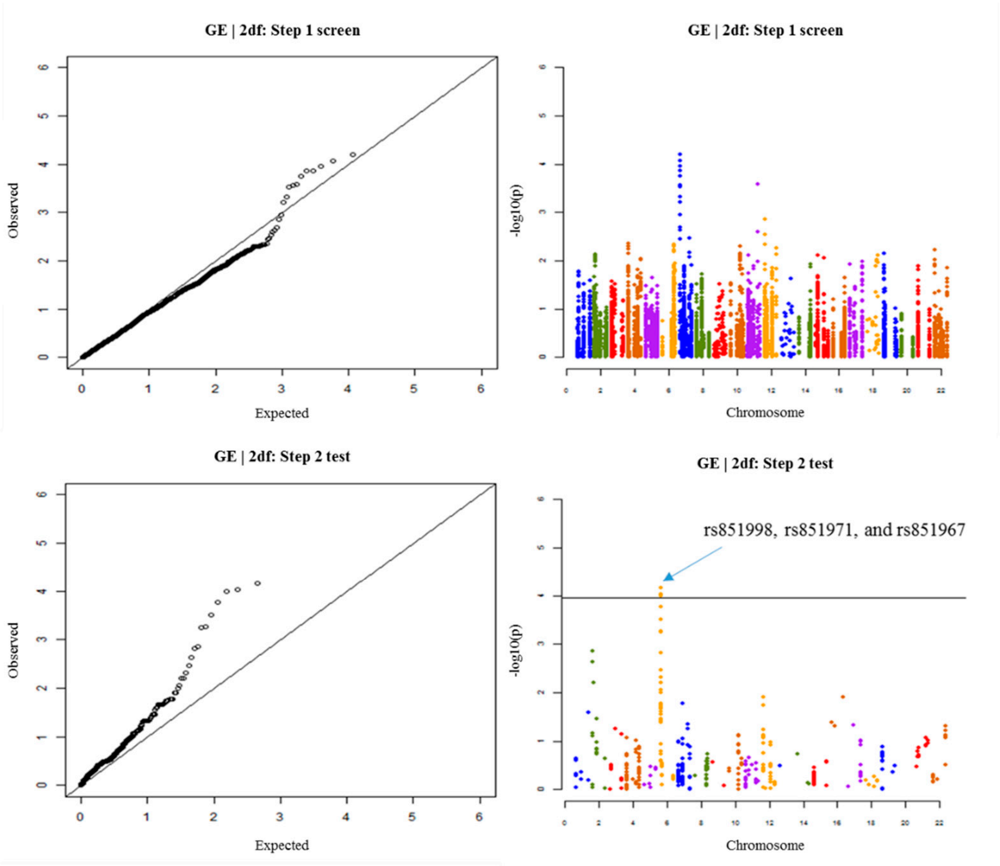

Figure S2. Result plot of GxE between 11,555 SNPs and height for breast cancer risk by usingGE|2df method.

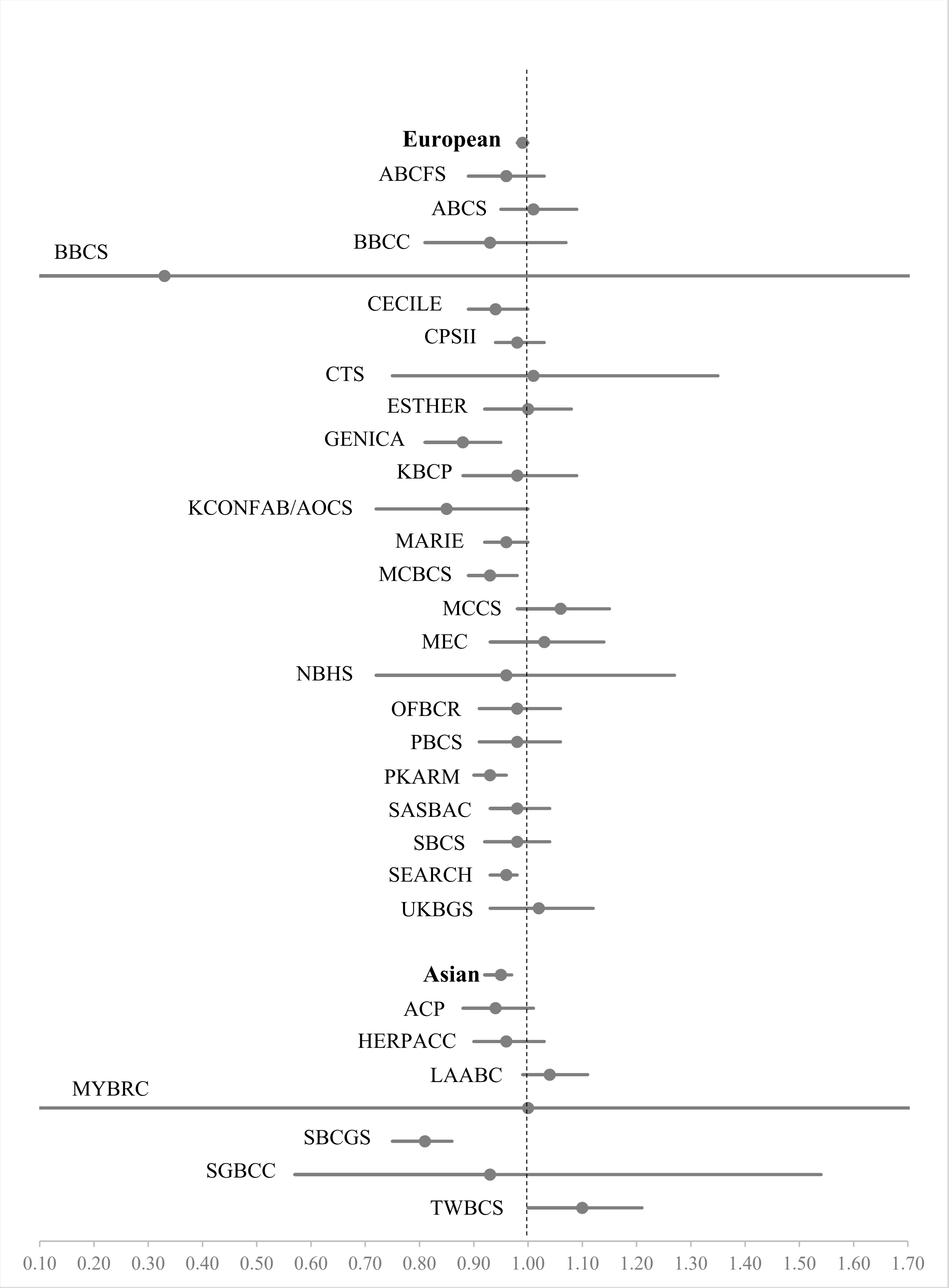

Figure S3. Forest plot presenting risk of age at menarche for breast cancer by study in BCAC.

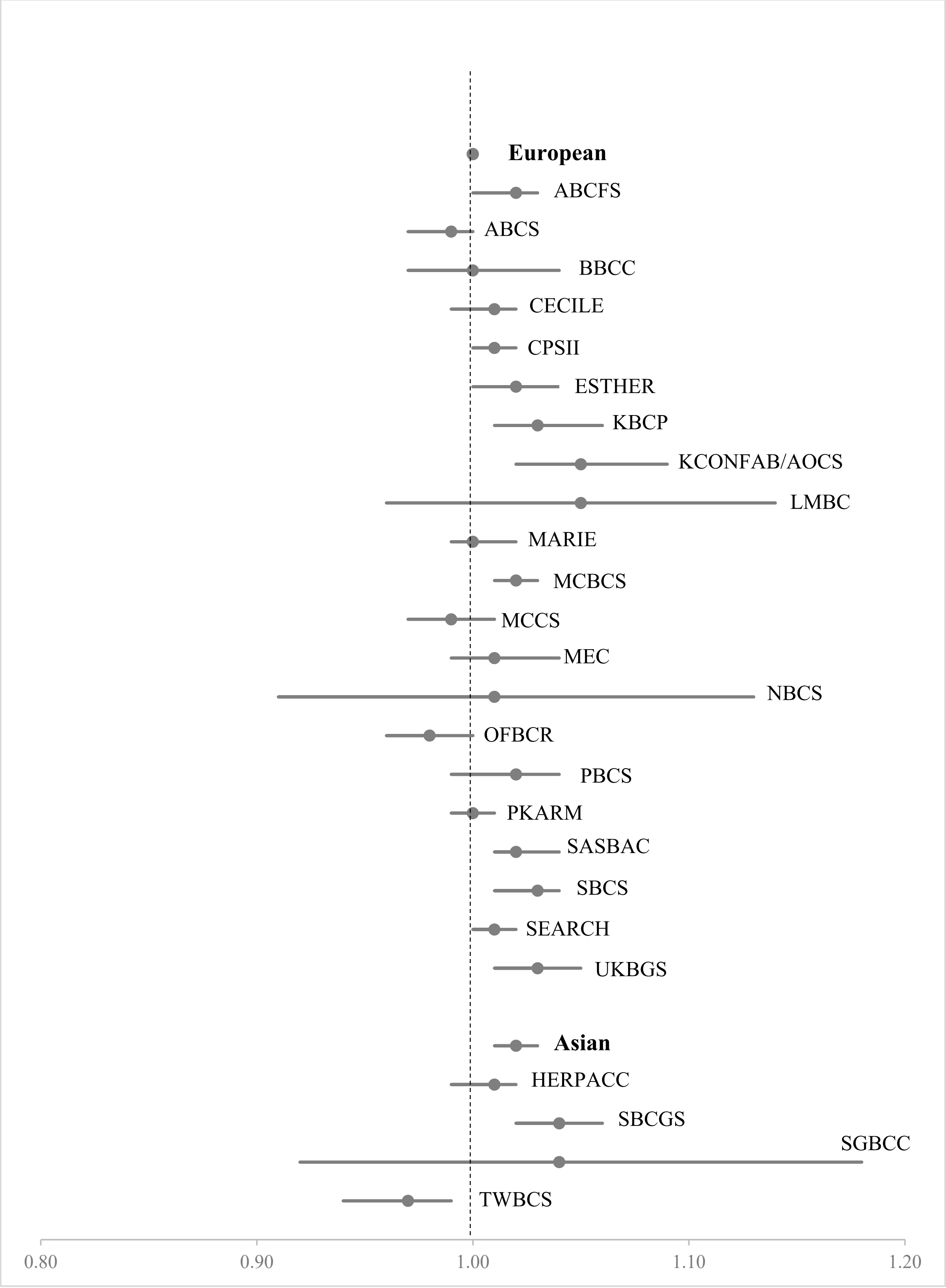

Figure S4. Forest plot presenting risk of height for breast cancer by study in BCAC.

Table S1. Distributions of demographic and reproductive factors and associations with the risk of breast cancer, Seoul Breast Cancer Study (Pre-imputation).

Demographic and reproductive factors	Cases		Controls			OR[c]	95% CI
	N = 1970		*N* = 2052		*p*		
	Mean ± SD	%	Mean ± SD	%			
Age	49.0 ± 8.26		51.4 ± 7.75		<0.0001	0.99	0.98–1.01
<40 years		11.3		0.1			
40–49 years		47.8		47.9			
50–59 years		27.9		35.0			
60–69 years		11.6		16.1			
70+ years		1.4		0.9			
Family history, yes		4.8		1.8	<0.0001	2.58	1.72–3.87
Height	157.9 ± 5.05		155.9 ± 5.08		<0.0001	1.07	1.06–1.09
BMI (kg/m^2)	23.2 ± 2.95		23.7 ± 3.00		<0.0001	0.98	0.95–1.00
Menarche age	14.8 ± 1.65		15.2 ± 1.82		<0.0001	0.90	0.87–0.94
Ever pregnancy		93.5		92.8	0.4026	0.19	0.10–0.34
Age at FFTP[a]	25.9 ± 3.49		25.1 ± 3.41		<0.0001	1.05	1.03–1.07
No. of children	2.2 ± 0.85		2.3 ± 0.91		0.0003	1.07	0.98–1.18
Never breastfeeding		22.3		14.5	<0.0001	1.28 [d]	1.07–1.54
Breastfeeding duration (months)	18.0 ± 22.59		24.0 ± 21.53		<0.0001	0.99 [d]	0.99–1.00
Breastfeeding duration per child (months)	7.3 ± 8.01		9.9 ± 7.27		<0.0001	0.97[d]	0.95–0.98
Duration of EEBF[b]	12.3 ± 6.22		10.5 ± 5.36		<0.0001	1.05 [e]	1.04–1.07
Postmenopausal women		38.2		54.8	<0.0001	0.56	0.46–0.67
Age at menopause	48.5 ± 5.40		49.2 ± 4.55		0.0058	0.97	0.94–0.99

[a] FFTP first full-term pregnancy (years); [b] EEBF estrogen exposure before the first full-term pregnancy (years); [c] ORs analyzed by logistic regression adjusting for age, family history, categorical FFTP, age at menarche, and menopausal status; [d] ORs analyzed by logistic regression adjusting for age, family history, categorical FFTP, age at menarche, menopausal status and the number of children; [e] ORs analyzed by logistic regression adjusting for age, family history, ever pregnancy and menopausal status.

Table S2. Distributions of demographic and reproductive factors in cases and controls by birth year groups, Seoul Breast Cancer Study (Imputed + binary).

Demographic and reproductive factors	Cases (*N* = 1970)						Controls (*N* = 2052)						
	1940 (*N* = 451)		1950 (*N* = 802)		1960 (*N* = 717)		1940 (*N* = 450)		1950 (*N* = 859)		1960 (*N* = 743)		*p*[a]
Age, mean (sd)	61.1	(4.8)	49.3	(3.0)	41.2	(3.3)	63.3	(3.7))	51.7	(2.8)	43.8	(2.12)	<0.0001
<40 years (%)	-		-		31.1		-		-		0.3		
40–49 years (%)	-		56.0		68.8		-		28.1		99.7		
50–59 years (%)	43.7		44.0		-		22.4		71.9		-		
60–69 years (%)	50.3		-		-		73.3		-		-		
70+ years (%)	6.0		-		-		4.2		-		-		
Family history, yes (%)	5.8		5.0		4.0		1.1		2.3		1.5		0.0580
Height(cm), mean (sd)	156.2	(5.1)	157.8	(4.9)	159.0	(4.9)	154.0	(5.1)	155.7	(4.9)	157.3	(4.7)	<0.0001
< 160cm (%)	69.8		59.5		53.3		87.6		77.2		66.9		
≥ 160cm (%)	30.2		40.5		46.7		12.4		22.8		33.1		
BMI (kg/m^2), mean (SD)	24.6	(2.9)	23.2	(2.8)	22.4	(2.8)	24.7	(2.9)	23.7	(2.9)	23.0	(2.9)	<0.0001
< 25 (%)	62.3		78.3		84.2		58.0		71.1		79.1		
≥ 25 (%)	37.7		21.7		15.8		42.0		28.9		20.9		
Menarche age, mean (SD)	15.6	(1.8)	14.9	(1.6)	14.1	(1.4)	16.1	(1.7)	15.3	(1.8)	14.7	(1.6)	<0.0001
≥ 14 years (%)	86.9		81.4		71.1		93.8		86.4		78.2		
<14 years (%)	13.1		18.6		28.9		6.2		13.6		21.8		
Ever pregnancy (%)	95.8		94.9		90.5		92.9		93.4		92.2		<0.0001
Age at FFTP[b], mean (SD)	25.1	(3.7)	25.7	(3.4)	26.8	(3.3)	24.4	(3.0)	24.9	(3.3)	25.8	(3.5)	<0.0001
<27 years (%)	66.7		59.1		44.6		70.2		70.7		59.1		
Nulliparity or ≥ 27 years (%)	33.3		40.9		55.4		29.8		29.3		40.9		
No. of children, mean (SD)	2.8	(1.2)	2.1	(0.7)	1.9	(0.6)	3.1	(1.2)	2.1	(0.7)	2.0	(0.6)	<0.0001
≥ 2 (%)	90.1		84.9		82.4		96.2		88.2		83.5		
<2 (%)	10.0		15.1		17.6		3.8		11.9		16.5		
Never breastfeeding (%)	12.8		19.1		32.3		8.5		11.9		21.3		<0.0001
Breastfeeding duration (months), mean (SD)	40.8	(30.8)	19.7	(15.2)	11.7	(11.4)	41.9	(28.5)	24.0	(16.9)	15.1	(12.6)	<0.0001
Breastfeeding duration per child (months), mean(SD)	11.2	(9.6)	7.2	(7.5)	4.1	(5.2)	12.4	(8.9)	10.6	(6.9)	7.3	(6.1)	<0.0001
≥2.5 month (%)	63.9		49.4		28.5		74.6		74.1		57.8		
<2.5 month (%)	36.1		50.6		71.5		25.4		25.9		42.2		
Duration of EEBF[c](years), mean (SD)	10.9	(8.2)	12.1	(6.6)	14.0	(5.7)	11.1	(10.4)	11.4	(7.9)	12.5	(6.2)	<0.0001
< 13 years (%)	74.3		68.1		44.9		88.9		79.2		65.0		
≥ 13 years (%)	25.7		31.9		55.1		11.1		20.8		35.0		
Postmenopausal women (%)	96.7		34.6		5.2		95.5		71.1		11.2		<0.0001
Age at menopause, mean (SD)	50.0	(4.9)	47.5	(4.8)	39.6	(3.5)	50.0	(4.6)	49.4	(3.7)	43.4	(3.5)	<0.0001

[a] p for trend (among control subjects) by generalized linear models adjusting for age, family history, ever pregnancy, age at FFTP, and age at menarche; [b] FFTP first full-term pregnancy (years); [c] EEBF estrogen exposure before the first full-term pregnancy (years).

Table S3. Studies including BCAC replication data set.

				Total		Data Set for Age at Menarche x NCOA1 (N = 71,532)			Data Set for Height x ESR1 (N = 62,152)		
**Ethnicity**	**Study Acronym**	**Study Name**	**Country**	***N***	**(Cases/Control)**	***N***	**(Cases/Controls)**	**%**	***N***	**(Cases/Controls)**	**%**
European	ABCFS	Australian Breast Cancer Family Study	Australia	1341	(790/551)	1282	(773/509)	1.8	1283	(777/506)	2.1
	ABCS	Amsterdam Breast Cancer Study	Netherlands	3900	(2085/1815)	1938	(778/1160)	2.7	1939	(779/1160)	3.1
	BBCC	Bavarian Breast Cancer Cases and Controls	Germany	1092	(634/458)	970	(595/375)	1.4	1079	(628/451)	1.7
	BBCS	British Breast Cancer Study	UK	2951	(1554/1397)	417	(206/211)	0.6	219	(5/214)	0.4
	CECILE	CECILE Breast Cancer Study	France	2018	(1019/999)	1987	(1004/983)	2.8	1914	(975/939)	3.1
	CGPS	Copenhagen General Population Study	Denmark	7482	(2948/4534)	1608	(1608/0)	2.3	2335	(2335/0)	3.8
	CNIO-BCS	Spanish National Cancer Centre Breast Cancer Study	Spain	1773	(902/871)	659	(5/654)	0.9	98	(0/98)	0.2
	CPSII	Cancer Prevention Study-II Nutrition Cohort	USA	4380	(2202/2178)	4330	(2173/2157)	6.1	4350	(2188/2162)	7.0
	CTS	California Teachers Study	USA	139	(68/71)	114	(60/54)	0.2	-	-	-
	ESTHER	ESTHER Breast Cancer Study	Germany	980	(478/502)	960	(467/493)	1.3	960	(471/489)	1.5
	GENICA	Gene Environment Interaction and Breast Cancer in Germany	Germany	892	(465/427)	890	(464/426)	1.2	892	(465/427)	1.4
	GLACIER	Study to Investigate the Genetics of Lobular Carcinoma In situ in Europe	UK	2322	(2322/0)	1894	(1894/0)	2.7	-	-	-
	HEBCS	Helsinki Breast Cancer Study	Finland	2898	(1664/1234)	362	(362/0)	0.5	345	(345/0)	0.6
	HMBCS	Hannover-Minsk Breast Cancer Study	Belarus	820	(690/130)	527	(527/0)	0.7	-	-	-
	ICICLE	Study to Investigate the Genetics of In Situ Carcinoma of the Ductal Subtype	UK	2902	(2902/0)	2777	(2777/0)	3.9	-	-	-
	KARBAC	Karolinska Breast Cancer Study	Sweden	1384	(722/662)	353	(353/0)	0.5	-	-	-
	KARMA	Karolinska Mammography Project for Risk Prediction of Breast Cancer – Cohort Study	Sweden	1	(1/0)	1	(1/0)	0.0	1	(1/0)	0.0
	KBCP	Kuopio Breast Cancer Project	Finland	696	(445/251)	656	(410/246)	0.9	661	(415/246)	1.1
	KCONFAB/AOCS	Kathleen Cuningham Foundation Consortium for research into Familial Breast Cancer/Australian Ovarian Cancer Study	Australia	1486	(588/898)	1047	(567/480)	1.5	1445	(578/867)	2.3
	LMBC	Leuven Multidisciplinary Breast Centre	Belgium	1802	(812/990)	1705	(1705/0)	2.4	2296	(1941/355)	3.7
	MARIE	Mammary Carcinoma Risk Factor Investigation	Germany	3596	(1818/1778)	3104	(1600/1504)	4.3	3594	(1816/1778)	5.8

	MBCSG	Milan Breast Cancer Study Group	Italy	888	(488/400)	177	(177/0)	0.3	177	(177/0)	0.3
	MCBCS	Mayo Clinic Breast Cancer Study	USA	3793	(1878/1915)	3327	(1671/1656)	4.7	3493	(1716/1777)	5.6
	MCCS	Melbourne Collaborative Cohort Study	Australia	1124	(690/494)	1116	(627/489)	1.6	1115	(626/489)	1.8
	MEC	Multiethnic Cohort	USA	1472	(742/730)	640	(298/342)	0.9	647	(300/347)	1.0
	NBCS	Norwegian Breast Cancer Study	Norway	1952	(1675/277)	37	(0/37)	0.1	280	(58/222)	0.5
	NBHS	Nashville Breast Health Study	USA	932	(562/320)	76	(36/40)	0.1	-	-	-
	OBCS	Oulu Breast Cancer Study	Finland	921	(507/414)	413	(413/0)	0.6	-	-	-
	OFBCR	Ontario Familial Breast Cancer Registry	Canada	1686	(1175/511)	1441	(952/489)	2.0	1443	(948/495)	2.3
	ORIGO	Leiden University Medical Centre Breast Cancer Study	Nether-lands	684	(357/327)	259	(259/0)	0.4	-	-	-
	PBCS	NCI Polish Breast Cancer Study	Poland	943	(519/424)	936	(515/421)	1.3	936	(514/422)	1.5
	PKARM	Karolinska Mammography Project for Risk Prediction of Breast Cancer – Case-Control Study	Sweden	11,001	(5537/5464)	10,274	(5228/5046)	14.4	10,552	(5334/5218)	17.0
	SASBAC	Singapore and Sweden Breast Cancer Study	Sweden	2541	(1163/1378)	2319	(1058/1261)	3.2	2523	(1153/1370)	4.1
	SBCS	Sheffield Breast Cancer Study	UK	1691	(843/848)	1658	(826/832)	2.3	1667	(827/840)	2.7
	SEARCH	Study of Epidemiology and Risk factors in Cancer Heredity	UK	17,407	(9338/8069)	11,281	(5971/5310)	15.8	9074	(6022/3052)	14.6
	SKKDKFZS	Städtisches Klinikum Karlsruhe Deutsches Krebsforschungszentrum Study	Germany	165	(136/29)	105	(105/0)	0.2	133	(133/0)	0.2
	UKBGS	UK Breakthrough Generations Study	UK	946	(482/464)	845	(429/416)	1.2	912	(467/445)	1.5
Asian	ACP	Asia Cancer Program	Thailand	1059	(423/636)	1053	(421/632)	1.5	-	-	-
	HERPACC	Hospital-based Epidemiologic Research Program at Aichi Cancer Center	Japan	2070	(694/1376)	2033	(686/1347)	2.8	2068	(694/1374)	3.3
	LAABC	Los Angeles County Asian-American Breast Cancer Case-Control Study	USA	1802	(812/990)	1800	(810/990)	2.5	-	-	-
	MYBRC	Malaysian Breast Cancer Genetic Study	Malaysia	1380	(770/610)	464	(291/173)	0.7	-	-	-
	SBCGS	Shanghai Breast Cancer Genetic Study	China	1740	(848/892)	1738	(847/891)	2.4	1739	(847/892)	2.8
	SGBCC	Singapore Breast Cancer Cohort	Singapore	1034	(532/502)	859	(472/387)	1.2	870	(462/408)	1.4
	TWBCS	Taiwanese Breast Cancer Study	Taiwan	1125	(889/236)	1100	(865/235)	1.5	1112	(876/236)	1.8

Table S4. Distributions of demographic and reproductive factors and associations with the risk of breast cancer by ethnicity, Breast Cancer Association Consortium replication dataset for age at menarche x NCOA1.

Demographic and reproductive factors	**European**						**Asian**							
	**Cases** *N* **= 36,894**		**Controls** *N* **= 25,591**				**Cases** *N* **= 4392**		**Controls** *N* **= 4655**					
	**Mean ± SD**	**%**	**Mean ± SD**	**%**	**OR[d]**	**95% CI**	**Mean ± SD**	**%**	**Mean ± SD**	**%**	**OR[d]**	**95% CI**	**I^2**	***p*-Value**
Age	57.8 ± 10.65		56.9 ± 10.41		1.02	1.02–1.02	52.7 ± 10.58		51.3 ± 10.44		1.03	1.02–1.03	58.7%	0.1195
Family history, yes		17.2		9.9	2.00	1.90–2.11		7.0	2.8	5.0	1.98	1.58–2.46	0.0%	0.9066
Missing		20.4		17.1				19.9	31.8					
Height	164.3 ± 6.60		164.5 ± 6.55		1.00	0.99–1.00	156.7 ± 5.91		156.3 ± 5.58		1.02	1.01–1.03	95.2%	<0.0001
< Q3[a]		60.4		60.8	1.00	reference		43.4		43.4	1.00	Reference		
≥ Q3[a]		19.7		21.0	0.96	0.92–1.00		21.5		18.1	1.23	1.09–1.39	93.0%	0.0002
Missing		19.9		18.3				35.1		38.6				
Menarche age	13.1 ± 1.58		13.1 ± 1.56		0.99	0.98–1.00	13.7 ± 1.80		13.8 ± 1.91		0.95	0.92–0.97	88.4%	0.0034
≥ Q1[b]		84.0		84.9	1.00	reference		92.3		91.7	1.00	Reference		
< Q1[b]		16.0		15.1	1.03	0.99–1.08		7.7		8.3	0.97	0.82–1.14	0.0%	0.4618
Ever pregnancy		82.6		85.6	0.77	0.73–0.81		84.6		83.0	0.78	0.69–0.89	0.0%	0.8554
Missing		2.9		2.4				1.2		5.2				
Age at FFTP	25.1 ± 4.88		25.1 ± 4.69		1.00	0.99–1.00	26.0 ± 4.50		25.3 ± 4.15		1.05	1.03–1.06	97.8%	<0.0001
<Q3[c]		52.5		58.9	1.00	reference		45.0		36.0	1.00	reference		
Nulliparity or ≥Q3[c]		35.2		33.8	1.17	1.13–1.22		38.1		25.1	1.14	1.03–1.27	0.0%	0.5944
Missing		12.3		7.2				17.0		38.9				
Postmenopausal women		62.9		68.6	0.67	0.64–0.70		36.5		36.6	0.83	0.73–0.95	89.8%	0.0018
Missing		8.7		2.2				24.3		21.8				

[a] 169 cm in European, and 160cm in Asian; [b] 12 years old in both European and Asian; [c] 28 years old in both European and Asian; [d] ORs analyzed by logistic regression adjusting for age, family history, categorical FFTP, age at menarche, and menopausal status; FFTP: first full-term pregnancy (years).

Table S5. Distributions of demographic and reproductive factors and associations with the risk of breast cancer by ethnicity, Breast Cancer Association Consortium replication dataset for height x ESR1.

Demographic and reproductive factors	**European**						**Asian**							
	**Cases** **N = 31,994**		**Controls** **N = 24,369**				**Cases** **N = 2879**		**Controls** **N = 2910**					
	**Mean ± SD**	**%**	**Mean ± SD**	**%**	**OR[d]**	**95% CI**	**Mean ± SD**	**%**	**Mean ± SD**	**%**	**OR[d]**	**95% CI**	**I^2**	***p*-Value**
Age	58.4 ± 11.00		56.2 ± 10.45		1.04	1.04–1.04	52.9 ± 10.48		51.6 ± 10.08		1.03	1.03–1.04	0.0%	0.4074
Family history, yes		19.1		9.9	1.74	1.65–1.83		8.9		4.0	1.74	1.37–2.23	0.0%	0.9784
Missing		9.8		25.0				2.1		11.5				
Height	164.3 ± 6.61		164.4 ± 6.62		1.00	1.00–1.00	156.7 ± 5.92		156.3 ± 5.60		1.02	1.01–1.03	94.5%	<0.0001
< Q3[a]		75.5		74.9	1.00	reference		67.0		70.9	1.00	Reference		
≥ Q3[a]		24.5		25.1	0.97	0.93–1.01		33.0		29.1	1.22	1.08–1.39	91.8%	0.0005
Menarche age	13.1 ± 1.56		13.1 ± 1.57		0.98	0.97–0.99	13.8 ± 1.71		13.9 ± 1.83		0.99	0.96–1.03	0.0%	0.5125
≥ Q1[b]		78.2		73.3	1.00	reference		76.6		74.4	1.00	Reference		
< Q1[b]		14.1		12.6	1.09	1.03–1.15		22.4		23.8	0.88	0.76–1.01	88.0%	0.0038
Missing		7.7		14.2				1.0		1.8				
Ever pregnancy		83.9		85.7	0.74	0.71–0.78		88.1		88.2	0.84	0.70–1.01	31.2%	0.2281
Missing		2.0		2.7				1.2		2.5				
Age at FFTP	25.0 ± 4.85		25.1 ± 4.78		1.00	0.99–1.00	26.4 ± 4.25		26.1 ± 3.72		1.03	1.01–1.04	91.5%	0.0006
<Q3[c]		56.4		53.6	1.00	reference		54.4		42.6	1.00	reference		
Nulliparity or ≥Q3[c]		35.8		31.8	1.14	1.10–1.19		42.0		28.5	1.24	1.10–1.40	33.1%	0.2213
Missing		7.8		14.5				3.6		28.9				
Postmenopausal women		67.1		63.4	0.57	0.54–0.60		44.0		47.2	0.80	0.67–0.95	92.8%	0.0002
Missing		3.4		10.6				8.6		0.9				

[a] 169 cm in European, and 160cm in Asian; [b] 12 years old in European and 13years old in Asian; [c] 28 years old in both European and Asian; [d] ORs analyzed by logistic regression adjusting for age, family history, categorical FFTP, age at menarche, and menopausal status; FFTP: first full-term pregnancy (years).

Table S6. Association between age at menarche and the risk of breast cancer in studies from BCAC that showed consistence with SeBCS.

Data Set for Age at Menarche x NCOA1				rs13035764		Age at Menarche									
**Study Acronym**	**Country**	***N***	**(Cases/Controls)**	**OR[a]**	**95% CI**	**Mean**	**SD**	**Q1**	**Median**	**Q3**	**OR[b]**	**95% CI**	**OR[c]**	**95% CI**	**p-GxE[d]**
CECILE	France	1987	(1004/983)	1.11	0.97–1.28	13.01	1.65	12	13	14	0.94	0.89–1.00	1.09	0.86–1.37	0.5696
GENICA	Germany	890	(464/426)	1.08	0.88–1.32	13.54	1.71	12	13	15	0.88	0.81–0.95	1.35	0.86–2.11	0.9250
KCONFAB/AOCS	Australia	1047	(567/480)	1.23	0.86–1.78	13.04	1.56	12	13	14	0.85	0.72–1.00	1.34	0.68–2.64	0.4994
MARIE	Germany	3104	(1600/1504)	1.06	0.95–1.18	13.59	1.68	12	14	15	0.96	0.92–1.00	1.01	0.79–1.29	0.4253
MCBCS	USA	3327	(1671/1656)	1.05	0.95–1.17	12.71	1.34	12	13	13	0.93	0.89–0.98	1.30	1.08–1.56	0.6289
PKARMA	Sweden	10,274	(5228/5046)	1.00	0.93–1.07	13.15	1.47	12	13	14	0.93	0.90–0.96	1.20	1.05–1.37	0.1263
SEARCH	UK	11,281	(5971/5310)	0.98	0.92–1.04	12.87	1.58	12	13	14	0.96	0.93–0.98	1.03	0.94–1.14	0.1359
SBCGS	China	1738	(847/891)	1.09	0.92–1.30	14.68	1.77	13	15	16	0.81	0.75–0.86	1.55	1.04–2.31	0.0890
	European*	31,910	(16,505/15,405)	1.00	0.97–1.04	13.05	1.56	12	13	14	0.95	0.94–0.97	1.10	1.04–1.17	0.2332
	Total#	33,648	(17,352/16,296)	0.99	0.96–1.02	13.13	1.61	12	13	14	0.96	0.94–0.97	1.09	1.03–1.16	0.2006

*Including CECILE, GENICA, KCONFAB/AOCS, MARIE, MCBCS, PKARMA, SEARCH; # European + SBCGS; [a] Risk of SNP for breast cancer; [b] Risk of age at menarche (continuous variable) for breast cancer; [c] Risk of age at menarche (binary variable; age at menarche < Q1) for breast cancer; [d] p for interaction between age at menarche (binary variable) and rs13035764.

Table S7. Association between height and the risk of breast cancer in studies from BCAC that showed consistence with SeBCS.

Data Set for Height x ESR1				rs851998		Height									
Study Acronym	Country	*N*	(Cases/Controls)	OR[a]	95% CI	Mean	SD	Q1	Median	Q3	OR[b]	95% CI	OR[c]	95% CI	p-GxE[d]
ABCFS	Australia	1283	(777/506)	0.92	0.77–1.11	163.8	6.85	158.00	163.00	168.00	1.02	1.00–1.03	1.32	1.03–1.68	0.2443
CPSII	USA	4350	(2188/2162)	1.00	0.92–1.10	164.2	6.32	160.02	165.10	167.64	1.01	1.00–1.02	1.09	0.96–1.24	0.7166
KBCP	Finland	661	(415/246)	1.24	0.98–1.58	161.7	6.00	158.00	162.00	165.00	1.03	1.01–1.06	1.66	1.15–2.39	0.6877
KCONFAB/AOCS	Australia	1445	(578/867)	1.06	0.73–1.53	163.5	6.93	157.48	163.00	168.00	1.05	1.02–1.09	1.39	0.81–2.41	0.5399
MCBCS	USA	3493	(1716/1777)	1.01	0.91–1.12	164.9	6.80	160.02	165.10	170.18	1.02	1.01–1.03	1.49	1.28–1.75	0.1401
SASBAC	Sweden	2523	(1153/1370)	1.00	0.89–1.13	164.0	5.55	160.00	164.00	168.00	1.02	1.01–1.04	1.33	1.10–1.59	0.0649
SBCS	UK	1667	(827/840)	0.95	0.82–1.11	161.9	6.72	157.00	163.00	168.00	1.03	1.01–1.04	1.50	1.18–1.90	0.6413
SEARCH	UK	9074	(6022/3052)	0.90	0.80–1.02	163.2	6.54	158.00	163.00	168.00	1.01	1.00–1.02	1.54	1.27–1.85	0.1017
UKBGS	UK	912	(467/445)	0.95	0.77–1.16	163.6	6.48	160.00	162.60	167.60	1.03	1.01–1.05	1.43	1.09–1.89	0.2281
SBCGS	China	1739	(847/892)	0.96	0.83–1.13	157.7	5.41	154.00	158.00	161.50	1.04	1.02–1.06	1.29	1.00–1.66	0.4982
	European*	25,408	(14,143/11,265)	0.99	0.95–1.03	163.63	6.53	160.00	163.00	168.00	1.01	1.01–1.02	1.29	1.22–1.38	0.6312
	Total#	27,147	(14,990/12,157)	0.97	0.94–1.01	163.25	6.63	158.00	163.00	168.00	1.01	1.01–1.02	1.33	1.25–1.41	0.4043

*Including ABCFS, CPSII, KBCP, KCONFAB/AOCS, MCBCS, SASBAC, SBCS, SEARCH, UKBGS; # European + SBCGS; [a] Risk of SNP for breast cancer; [b] Risk of height (continuous variable) for breast cancer; [c] Risk of height (binary variable; age at height < Q1) for breast cancer; [d] p for interaction between height (binary variable) and rs851998.

Table S8. Association between environmental factor and breast cancer by genotypes according to ER/PR status, Seoul Breast Cancer Study.

Exposure	Category	Total			ER+			ER-			Heterogeneity	
		N(Cases/Controls)	OR	95% CI	N(Cases/Controls)	OR	95% CI	N(Cases/Controls)	OR	95% CI	I²	*p*-Value
Age at menarche < 14 years old	Overall	4022(1970/2052)	1.27	1.07–1.50	3262(1210/2052)	1.27	1.05–1.54	2764(712/2052)	1.27	1.02–1.59	0%	1.0000
	E ∣ rs13035764_CC	1963(1007/956)	1.19	0.95–1.51	1576(620/956)	1.24	0.96–1.61	1315(359/956)	1.12	0.82–1.53	0%	0.8867
	E ∣ rs13035764_CG	1706(810/896)	1.17	0.89–1.53	1384(488/896)	1.09	0.77–1.46	1201(305/896)	1.28	0.90–1.82	0%	0.7887
	E ∣ rs13035764_GG	337(147/190)	2.85	1.43–5.67	290(100/190)	3.08	1.47–6.47	234(44/190)	2.76	1.11–6.92	0%	0.9808
Height ≥ 160cm	Marginal	4022(1970/2052)	1.92	1.67–2.20	3262(1210/2052)	1.90	1.62–2.22	2764(712/2052)	1.86	1.54–2.24	0%	0.9642
	E ∣ rs851998_GG	1281(677/604)	1.53	1.20–1.95	1011(407/604)	1.52	1.15–2.01	863(259/604)	1.49	1.09–2.05	0%	0.9915
	E ∣ rs851998_GA	1921(938/983)	2.03	1.65–2.49	1573(590/983)	1.96	1.56–2.47	1306(323/983)	2.00	1.52–2.64	0%	0.9755
	E ∣ rs851998_AA	819(355/464)	2.39	1.74–3.29	677(213/464)	2.51	1.74–3.63	594(130/464)	2.27	1.46–3.53	0%	0.9425

Exposure	Category	Total			PR+			PR-			Heterogeneity	
		N(Cases/Controls)	OR	95% CI	N(Cases/Controls)	OR	95% CI	N(Cases/Controls)	OR	95% CI	I²	*p*-Value
Age at menarche < 14years old	Overall	4022(1970/2052)	1.27	1.07–1.50	3126(1074/2052)	1.22	1.00–1.50	3941(1955/1986)	1.34	1.09–1.66	0%	0.8254
	E ∣ rs13035764_CC	1963(1007/956)	1.19	0.95–1.51	1512(556/956)	1.18	0.90–1.56	1378(422/956)	1.26	0.94–1.68	0%	0.9396
	E ∣ rs13035764_CG	1706(810/896)	1.17	0.89–1.53	1326(430/896)	1.03	0.74–1.44	1257(361/896)	1.26	0.90–1.77	0%	0.7017
	E ∣ rs13035764_GG	337(147/190)	2.85	1.43–5.67	275(85/190)	3.19	1.48–6.85	251(61/190)	2.57	1.09–6.06	0%	0.9336
Height ≥ 160cm	Marginal	4022(1970/2052)	1.92	1.67–2.20	3126(1074/2052)	2.05	1.74–2.42	2899(847/2052)	1.70	1.42–2.03	14.4%	0.3108
	E ∣ rs851998_GG	1281(677/604)	1.53	1.20–1.95	979(375/604)	1.62	1.22–2.17	895(291/604)	1.37	1.01–1.87	0%	0.7374
	E ∣ rs851998_GA	1921(938/983)	2.03	1.65–2.49	1503(520/983)	2.16	1.70–2.75	1375(392/983)	1.73	1.33–2.24	0%	0.4484
	E ∣ rs851998_AA	819(355/464)	2.39	1.74–3.29	643(179/464)	2.66	1.80–3.93	628(164/464)	2.29	1.53–3.44	0%	0.8618

ORs analyzed by logistic regression adjusting for age, family history, categorical FFTP, age at menarche, and menopausal status.
